# Carbon steel corrosion by bacteria from failed seal rings at an offshore facility

**DOI:** 10.1038/s41598-020-69292-5

**Published:** 2020-07-23

**Authors:** Silvia J. Salgar-Chaparro, Adam Darwin, Anna H. Kaksonen, Laura L. Machuca

**Affiliations:** 1 Curtin Corrosion Centre, WA School of Mines: Minerals, Energy and Chemical Engineering, Curtin University, Kent Street, Bentley, WA 6102 Australia; 2 Woodside Energy Ltd., Perth, WA 6000 Australia; 3 Commonwealth Scientific and Industrial Research Organization (CSIRO), Land and Water, 147 Underwood Avenue, Floreat, WA 6014 Australia

**Keywords:** Microbial communities, Corrosion, Metals and alloys, Biofilms

## Abstract

Corrosion of carbon steel by microorganisms recovered from corroded seal rings at an offshore floating production facility was investigated. Microbial diversity profiling revealed that communities in all sampled seal rings were dominated by *Pseudomonas* genus. Nine bacterial species, *Pseudomonas aeruginosa* CCC-IOB1*, Pseudomonas balearica* CCC-IOB3*, Pseudomonas stutzeri* CCC-IOB10*, Citrobacter youngae* CCC-IOB9*, Petrotoga mobilis* CCC-SPP15*, Enterobacter roggenkampii* CCC-SPP14*, Enterobacter cloacae* CCC-APB1*, Cronobacter sakazakii* CCC-APB3*,* and *Shewanella chilikensis* CCC-APB5 were isolated from corrosion products and identified based on 16S rRNA gene sequence. Corrosion rates induced by the individual isolates were evaluated in artificial seawater using short term immersion experiments at 40 °C under anaerobic conditions. *P. balearica, E. roggenkampii,* and *S. chilikensis,* which have not been associated with microbiologically influenced corrosion before, were further investigated at longer exposure times to better understand their effects on corrosion of carbon steel, using a combination of microbiological and surface analysis techniques. The results demonstrated that all bacterial isolates triggered general and localised corrosion of carbon steel. Differences observed in the surface deterioration pattern by the different bacterial isolates indicated variations in the corrosion reactions and mechanisms promoted by each isolate.

## Introduction

Corrosion is a ubiquitous problem that affects almost all industrial sectors including oil and gas production, transportation and refining facilities^1,2^, mining^3^, marine engineering and shipping^4,5^, industrial water systems^6^, food processing plants^7^, nuclear industries^8^, among others. This phenomenon occurs via electrochemical reactions, where electrons are released from the metal at anodic sites and are gained at cathodic sites^9^. Although assessment of the cost of corrosion is difficult, the NACE International IMPACT study estimated the global cost of corrosion as US$2.5 trillion in 2013^10^. Microbiologically influenced corrosion (MIC) has been estimated to contribute at least 20% to 40% of the total corrosion costs^11,12^. The loss of integrity of industrial infrastructure can result in substantial economic, environmental, health, safety and technological consequences^13^.

MIC is a type of corrosion in which the deterioration of metals occurs due to the presence and activity of microorganisms^14^. Microorganisms initiate, facilitate or accelerate corrosion reactions by altering the electrochemical conditions in the metal-solution interface^15^. Compared to other forms of corrosion, MIC is highly unpredictable and occurs at rates as high as 10 mm year^−1^^16^. Early detection of MIC is difficult due to its localised nature and the wide range of environmental conditions and associated microorganisms^17^. MIC has been proposed as the cause of failure in many significant incidents in the hydrocarbon industry such as the propane tank leak and explosion in Umm Said NGL Plant (Qatar)^18^, the natural gas leak and explosion in Carlsbad (New Mexico)^19,20^, and the oil spill in the Prudhoe Bay oil field (Alaska North Slope)^21^.

Microorganisms promote corrosion through their metabolic activities, and several mechanisms have been proposed in the literature to explain MIC. The main mechanisms include the formation of concentration cells, the production of corrosive metabolites, the dissolution of protective films, the creation of unprotective surface layers, and the uptake of electrons directly from the metal^22^. Various microorganisms have been implicated in MIC; these are commonly categorised in groups according to their metabolic capabilities. Main groups related with MIC include sulphate-reducing bacteria and archaea^23,24^, thiosulphate-reducing bacteria^25^, acid-producing bacteria^26^, iron-oxidising bacteria^27^, iron-reducing bacteria^28^, nitrate-reducing bacteria^29^, and methanogenic archaea^30^. Therefore, MIC is a complex phenomenon that can be triggered by several microorganisms with different metabolic capabilities.

Prevention of MIC in the industry requires the control of the growth and activity of microorganisms, which, provided the right environmental conditions exist, can thrive and initiate or accelerate corrosion of equipment and pipelines. Biocide treatment is the traditional method used in the oil and gas industry to mitigate MIC. However, there remain challenges associated with MIC mitigation, mainly due to the formation of recalcitrant biofilms, where antimicrobial compounds are known to be less effective^31^. Lower biocide efficacy has been attributed to the diffusion barrier generated by the biofilm structure, which results in cells close to the metal surface being exposed to sublethal biocide concentrations and the consequent development of biofilm resistance^32^. When biofilms have formed, MIC mitigation becomes a challenge to corrosion engineers; corrosion failures due to MIC can occur despite the application of biocide treatment^33–36^.

Although MIC has been studied for many decades^37^, fundamental knowledge gaps persist in the understanding of the processes leading to MIC on steel materials. Several laboratory studies have included corrosion experiments with reference strains^38,39^, or strains isolated from various environments^25,40^. However, only a few corrosion studies have been conducted using microorganisms recovered from corroded equipment. Since microorganisms are characterised by high genetic diversity and genotypic variation occurs as a result of environmental adaptation^41,42^, the isolation and study of microorganisms from corroded equipment provide a unique opportunity to expand our knowledge of MIC through the investigation of novel species implicated in corrosion, their metabolic capabilities and their potential role in corrosion.

In oil and gas pipelines and all piping systems, a pressure test called hydrostatic testing is normally carried out to inspect the system for leaks, to evaluate its integrity, and to validate that the system can operate under desired conditions^2,43^. During hydrostatic testing carried out on a floating production storage and offloading (FPSO) facility located on the Australian North West Shelf, several leaks were detected at piping system seal rings. These rings were exposed to production fluids during regular operation (~ 80 °C) for over five years. However, the system had been depressurised, flushed and filled with seawater (previously treated with oxygen scavenger and biocide) to conduct a hydrostatic test. This treated seawater was left in place for about four (4) months before hydrotesting was performed. The present investigation describes the microbial community identified in the corroded seal rings, as well as the isolation and identification of cultivable species within the community. The ability of the isolates to instigate MIC of carbon steel is examined and discussed.

## Results

### Microbial community composition in corrosion products from failed seal rings

**Figure 1:**
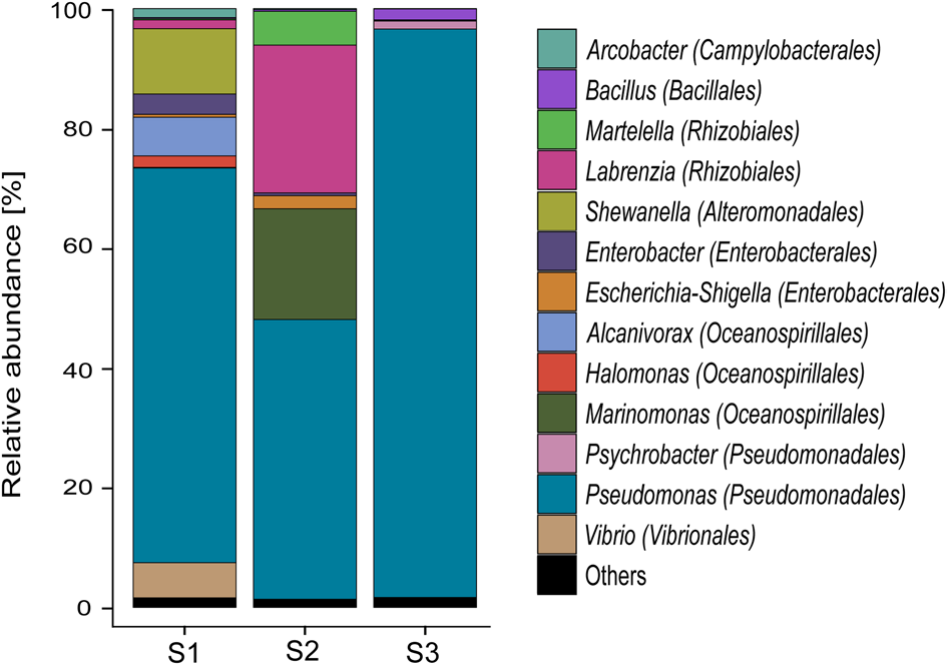
Microbial community composition of failed seal rings at the genus level based on partial 16S rRNA gene sequencing. Phylogenetic order is indicated in parentheses. Genera with relative abundances lower than 1% in all samples were summarised in the artificial group “Others”. S1: Sample 1; S2: Sample 2; S3: Sample 3. Figure generated using ggplot2 version 3.1.0 (R package). https://cran.r-project.org/web/packages/ggplot2/.

Microbial community composition in corroded seal rings was investigated with 16S rRNA gene sequencing. Miseq sequencing generated a total of 789,041 raw-reads, however, after the bioinformatics processing (quality filtering, singletons, and chimera removal) only 275,501 high-quality sequences were used for the taxonomic classification of the microorganisms present in the three collected samples. Data analysis indicated that all sequences belonged to the Bacteria domain, most of them affiliated to the *Proteobacteria* phylum (~ 98% relative abundance). Other phyla detected in lower abundance included *Firmicutes*, *Bacteroidetes, Cyanobacteria,* and *Epsilonbacteraeota*. Taxonomic classification with Silva 132 database showed the presence of 37 different genera in the microbial communities. The genera with relative abundances equal or greater to 1% in at least one of the samples are presented in Fig. 1. The complete list of microorganisms identified in the microbial community of corroded seal rings is given in Table S1.

DNA-based diversity profiling revealed differences in the microbial community composition among samples. Higher diversity was observed in sample 1 (Shannon index = 3.7) and lower diversity in sample 3 (Shannon index 2.1). Despite the differences observed in the relative abundances of dominant genera across samples, the microbial communities in all sampled seal rings were dominated by *the Pseudomonas* genus. Although the production fluid in the offshore facility is transported at high temperature (~ 80 °C), none of the genera detected has been classified as thermophilic microorganism. Conversely, all genera identified in the samples have been classified as mesophiles, and most of them have also been associated with marine environments and hydrocarbon degradation (*Martelella, Shewanella, Alcanivorax, Halomonas,* and *Marinomonas).* Most of the microorganisms detected in the seal rings are classified as aerobic species, although many of them can survive without oxygen (facultative anaerobes).

### Isolation, molecular identification and phylogenetic analysis

The three culture media used for the cultivation of sulphide producing prokaryotes (SPP), acid-producing bacteria (APB) and iron-oxidising bacteria (IOB) exhibited positive growth after four weeks of incubation. Nine different colony morphologies were observed on solid media, and all of the strains were rod-shaped Gram-negative bacteria (Table S2). Colonies were transferred at least three times to the same medium for complete purification. Comparison of the nucleotide sequences of the 16S rRNA gene with previously published sequences in the National Center of Biotechnology Information (NCBI) allowed the identification of the isolated bacteria to species level. The similarities and species identified by BLASTn analysis are given in Table 1. Three of the nine isolated microorganisms were classified into the *Pseudomonas* genus, which was found to be the most abundant genus in the microbial communities identified in corroded seal rings (Fig. 1). Other genera found as part of the microbial community and that were cultivated in the laboratory were *Enterobacter* and *Shewanella*. Bacteria belonging to the genera *Cronobacter, Petrotoga,* and *Citrobacter* were isolated from the seal rings even though they were not detected through DNA analysis, possibly because they were present in low abundances.

**Table 1:** Identification of bacteria isolated from corroded seal rings using 16S rRNA gene sequences.

Isolate ID	Accession no.	Best match in GenBank database (Accession no.)	Similarity %
CCC-APB1	MT215169	*Enterobacter cloacae* subsp. *cloacae* strain ATCC 1,304 (CP001918.1)	99.84
CCC-APB3	MT215170	*Cronobacter sakazakii* strain ATCC 29,544 (NR_118449.1)	99.7
CCC-APB5	MT215171	*Shewanella chilikensis* strain JC5 (NR_117772.1)	99.78
CCC-SPP14	MT215176	*Enterobacter roggenkampii* strain DSM 16,690 (CP017184.1)	99.93
CCC-SPP15	MT215177	*Petrotoga mobilis* strain SJ95 (CP000879.1)	99.84
CCC-IOB1	MT215172	*Pseudomonas aeruginosa* strain JCM 5,962 (MK796437.1)	100
CCC-IOB3	MT215173	*Pseudomonas balearica* strain SP1402 (NR_025972.1)	99.71
CCC-IOB9	MT215174	*Citrobacter youngae* strain NCTC13709 (LR134485.1)	99.42
CCC-IOB10	MT215175	*Pseudomonas stutzeri* strain ATCC 17,588 = LMG 11,199 (MT027239.1)	100

**Figure 2:**
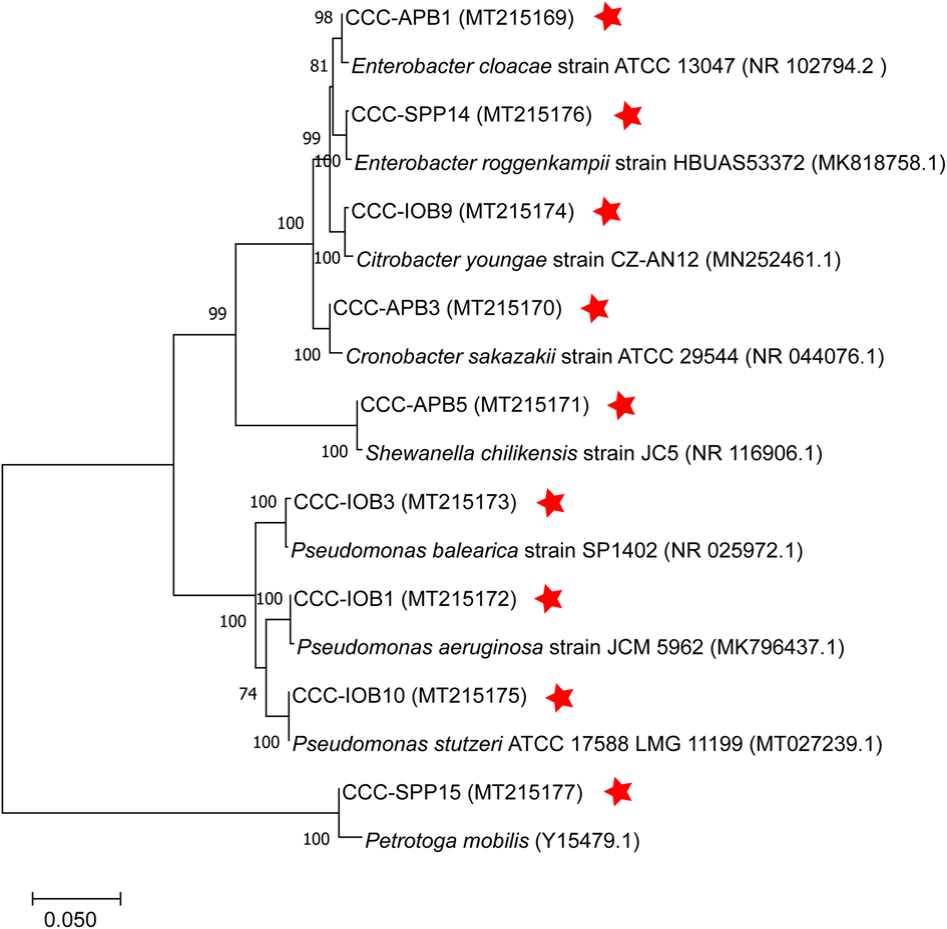
Neighbour-joining tree based on partial 16S rRNA gene sequences, showing phylogenetic relationships between isolates and related species. The percentage of replicate trees in which the associated taxa clustered together in the bootstrap test (1,000 replicates) is displayed next to the branches. The tree is drawn to scale, with branch lengths in the same units as those of the evolutionary distances used to infer the phylogenetic tree. GenBank accession numbers are given in parentheses. Red star indicates bacteria isolated in this study. Figure generated using MEGAX. https://www.megasoftware.net/.

A phylogenetic tree was constructed to analyse the relationships among the sequences of the isolated species and sequences from related organisms available in the GenBank database. The optimal tree with the sum of branch length = 6.61426233 is shown in Fig. 2. The percentage of replicate trees in which the associated taxa clustered together in the bootstrap test (1,000 replicates) are shown next to the branches. The tree is drawn to scale, with branch lengths in the same units as those of the evolutionary distances used to infer the phylogenetic tree.

### Microbiologically influenced corrosion

#### Corrosion screening test

**Figure 3:**
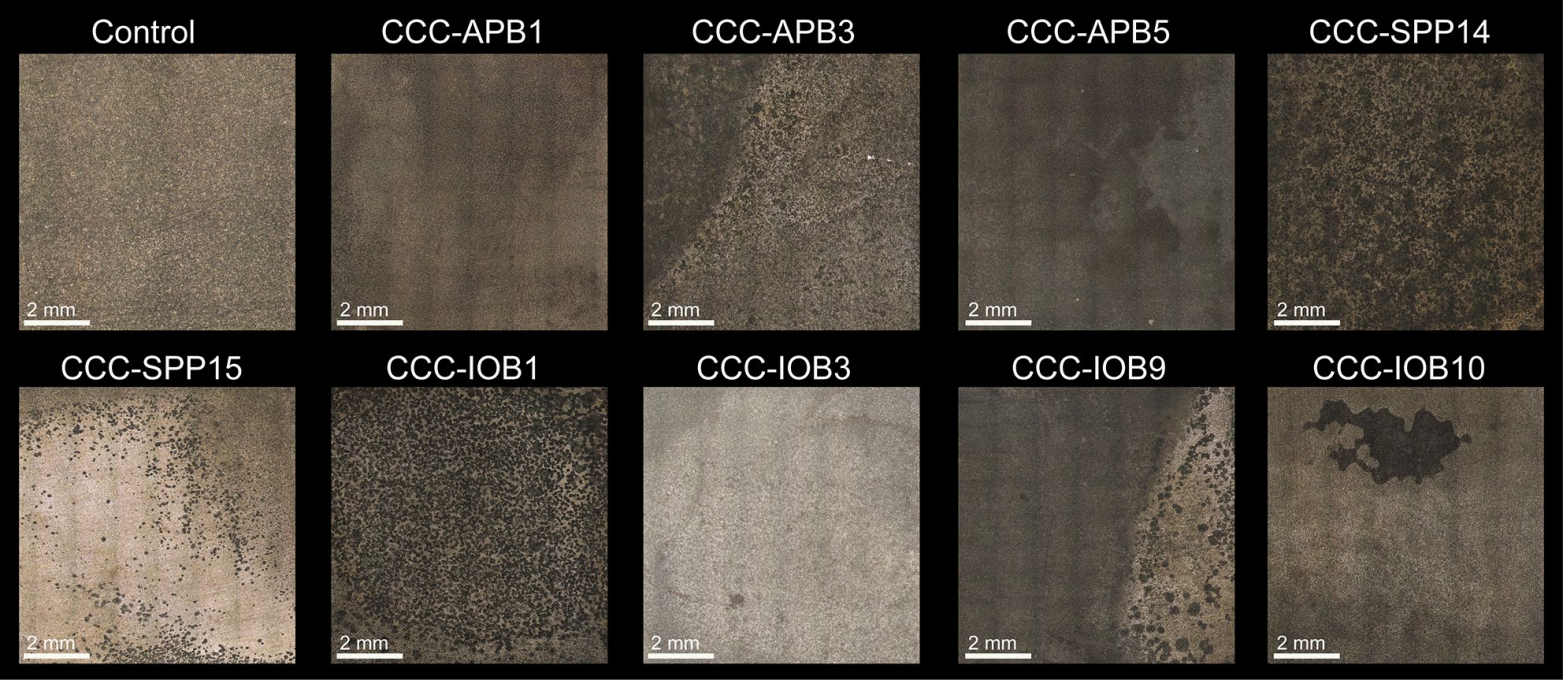
Microscope images of the surfaces of carbon steel coupons exposed for 7 days to bacterial isolates recovered from corroded seal rings. Control: coupon exposed to sterile test solution without bacteria. Images taken with the Alicona InfiniteFocus G4 at a lens magnification of ×5 (IFM version 3.5).

**Figure 4:**
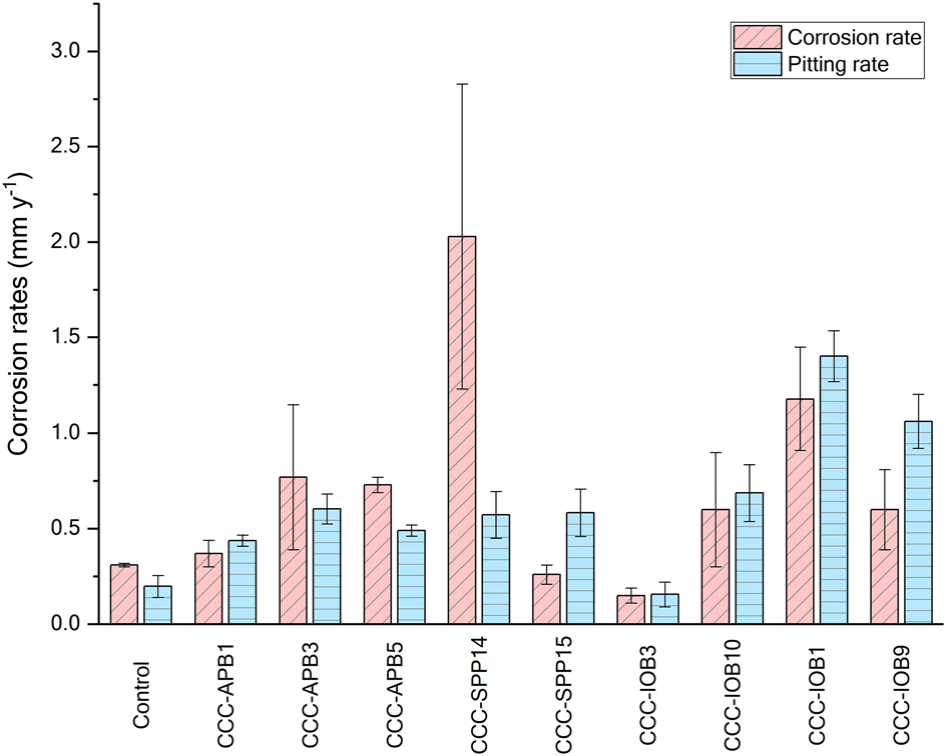
Corrosion rates of carbon steel during exposure to bacteria isolates for 7 days. Corrosion rate was calculated from the average weight loss of three coupons. Pitting rate was calculated from the maximum pit depth found in each replicate. Figure generated using OriginPro version 2020 (OriginLab Corporation). https://www.originlab.com/.

Carbon steel was exposed to isolated bacteria for 7 days to characterise their corrosive behaviour under anaerobic conditions. Surface profilometry analysis (Fig. 3) showed that in the presence of bacterial isolates except for CCC-IOB3 (*P. balearica*), the metal surface exhibited greater deterioration compared to the abiotic control. The weight loss in each coupon after exposure to abiotic and biotic conditions is shown in Table S3. The corrosion rate and pitting rate calculated from the weight loss and maximum pitting depth, respectively, measured for each isolate are shown in Fig. 4. Most of the isolates significantly increased the corrosion rates and pitting rates of carbon steel coupons as compared to abiotic control (Table S4). The corrosion rates triggered by the isolated bacteria showed distinct trends among species. Isolates such as CCC-APB3 (*C. sakazakii*), CCC-APB5 (*S. chilikensis*), and CCC-SPP14 (*E. roggenkampii*) induced higher general corrosion rates than pitting rates. In contrast, the isolates CCC-APB1 (*E. cloacae*), CCC-SPP15 (*P. mobilis*), CCC-IOB1 (*P. aeruginosa*), CCC-IOB9 (*C. youngae*), CCC-IOB10 (*P. stutzeri*) induced higher pitting rates than general corrosion rates. Different to all other isolates, carbon steel exposure to CCC-IOB3 (*P. balearica*) resulted in corrosion inhibition. Optical microscopy revealed that after 7 days of carbon steel exposure to *P. balearica*, the metal surface was completely covered by a biofilm layer. Instead, for the other isolates, carbon steel was covered by patchy biofilms (Fig. S1). The corrosion rates influenced by the isolated bacteria were classified from low to severe (Table S5), according to NACE SP0775 standard^44^.

#### MIC studies with selected bacterial isolates

Three of the isolated bacteria were selected for further corrosion investigation. The selection was based on the results of the corrosion screening test and the importance of studying microorganisms associated with different microbial groups. Microorganisms selected were CCC-IOB3 (*P. balearica*), CCC-SPP14 (*E. roggenkampii*), and CCC-APB5 (*S. chilikensis*). The three isolates belong to genera that have been previously associated with MIC; however, none of the specific species identified has been related to MIC before. Corrosion experiments consisted of exposing carbon steel coupons to active cultures of each isolate for 21 days. During the immersion period, planktonic cell numbers and solution pH were frequently monitored (Fig. S2). It was evident that the metabolic activities of the isolates altered the pH of the solution. *P. balearica* and *S. chilikensis* increased the pH, whereas *E. roggenkampii* decreased it. Meanwhile, the pH of the control test without bacteria remained stable during the test. The growth pattern of planktonic cells in the reactors as estimated by microscopic counting is shown in Fig. S2. The fluctuation in the number of cells detected throughout exposure was influenced by the solution replenishment that removed part of the population every 3 days. However, at the end of the test, the three isolates reached similar cell numbers in the bulk medium. On the other hand, the numbers of cells estimated to be attached to the coupons (sessile) at the end of the immersion period based on quantitative polymerase chain reaction (qPCR) varied among isolates. The highest cell density was reached by *S. chilikensis* (2.96 × 10^9^ cell cm^−2^), followed by *E. roggenkampii* (1.73 × 10^8^ cell cm^−2^) and finally, by *P. balearica* (1.45 × 10^5^ cell cm^−2^). At the end of the test, nitrites were detected in the bulk test solution of the three biotic reactors, which indicated that all isolates reduced nitrate. However, the presence of H_2_S was only detected in the reactor with *S. chilikensis.* Detection of sulphide and blackening of the test solution indicated that this isolate also reduced thiosulphate.

**Figure 5:**
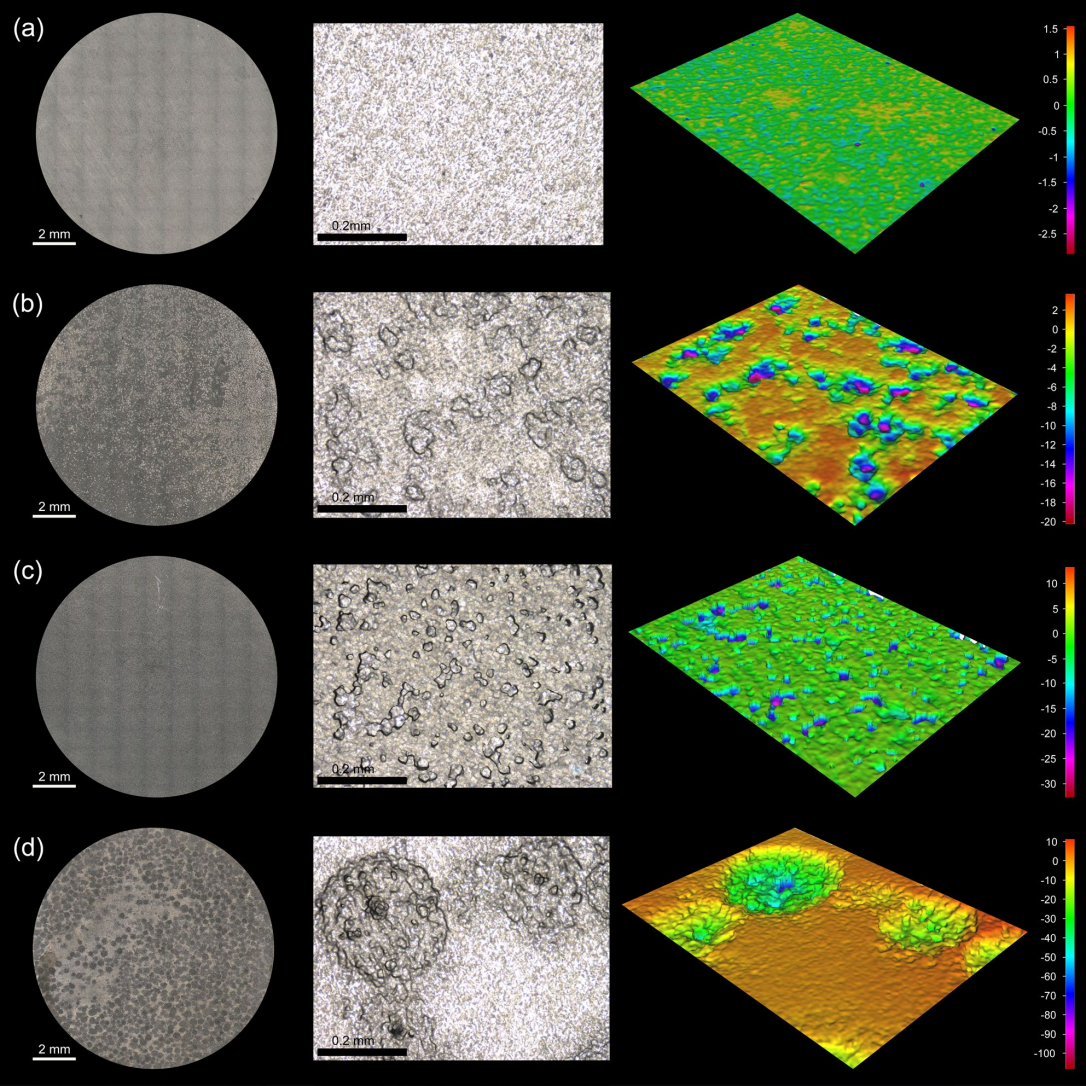
Microscope images of the surfaces of carbon steel coupons exposed for 21 days to (**a**) control (**b**) *P. balearica* CCC-IOB3 (**c**) *E. roggenkampii* CCC-SPP14 (**d**) *S. chilikensis* CCC-APB5. 2D image of the total surface (left image) and 3D images of 0.38 mm^2^ of the coupon surface (middle and right image). Images taken with the Alicona InfiniteFocus G4 at a lens magnification of × 5 and × 20 (IFM version 3.5).

**Figure 6:**
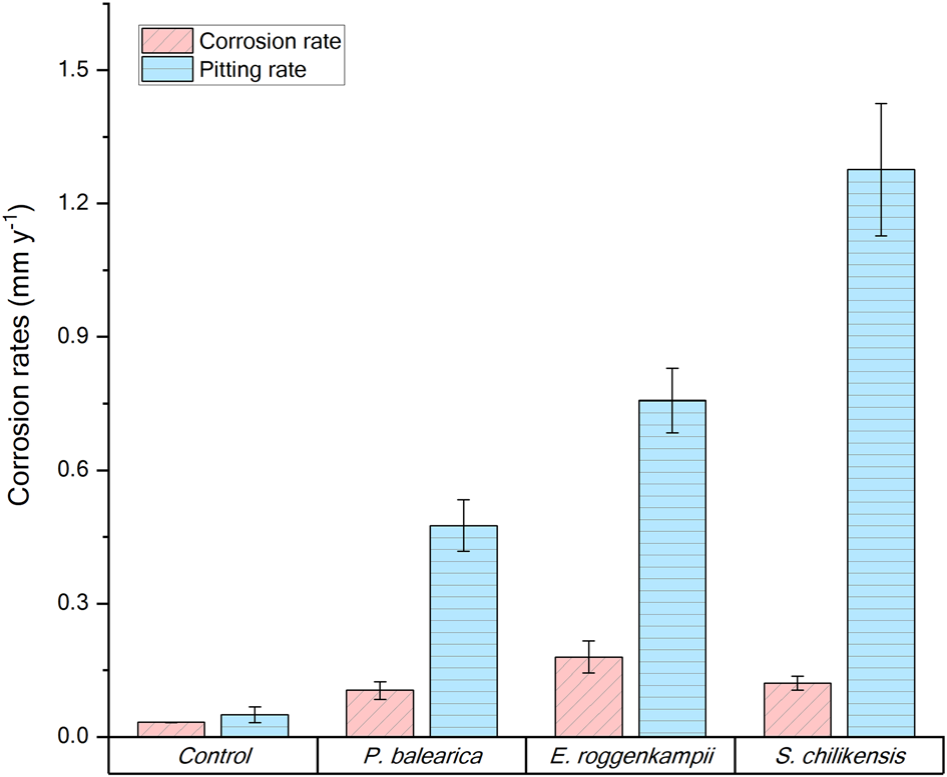
Corrosion rates of carbon steel after exposure to bacterial isolates for 21 days in the reactor experiment. Corrosion rate was calculated from the average weight loss of three coupons. Pitting rate was calculated from the maximum pit depth found in each replicate. Figure generated using OriginPro version 2020 (OriginLab Corporation). https://www.originlab.com/.

After retrieval of coupons from reactors, differences in the metal surface were observed among the tests. Coupons exposed to the isolates were covered by layers of corrosion products and cells that varied in colour and thickness. The surface analysis showed differences in deterioration and morphology of damage for the various isolates (Fig. 5). It was noted that all isolates caused MIC, including the *P. balearica* isolate that had resulted in corrosion inhibition in the short term corrosion test. Results of the corrosion measurements indicated that *P. balearica, E. roggenkampii* and *S. chilikensis* promoted MIC under the studied conditions (Fig. 6). The weight loss in coupons exposed to abiotic and biotic conditions is shown in Table S6. According to the Kruskal–Wallis test, there were significant differences in the corrosion rates and pit depth among coupons exposed to the three isolates and the control (*p* < 0.05). Isolates were shown to increase the corrosion rate and pitting rate of carbon steel (Table S7). Coupons exposed to *E. roggenkampii* exhibited the highest corrosion rate, whereas coupons exposed to *S. chilikensis* exhibited the deepest pits*.*

**Figure 7:**
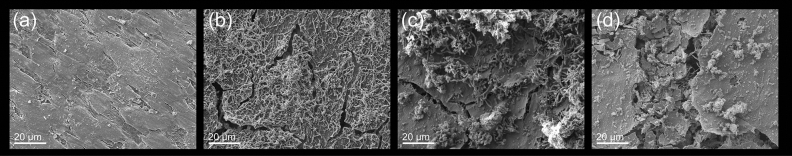
FESEM views of the biofilms/corrosion products formed over carbon steel coupons. (**a**) Control (**b**) *P. balearica* CCC-IOB3 (**c**) *E. roggenkampii* CCC-SPP14 (**d**) *S. chilikensis* CCC-APB5. Images taken with a Zeiss Neon Dual-Beam FESEM.

**Figure 8:**
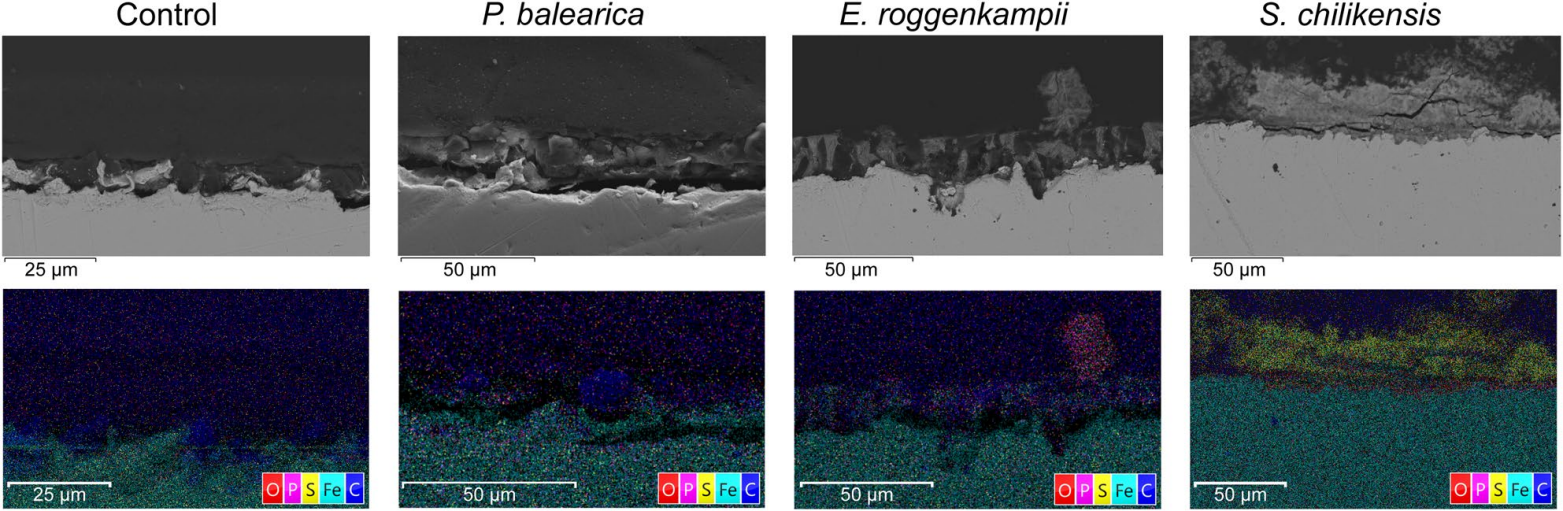
FESEM image and EDS-elemental mapping of cross-sectioned coupons exposed to the control and three bacterial species *P. balearica* CCC-IOB3, *E. roggenkampii* CCC-SPP14, and *S. chilikensis* CCC-APB5. Images generated using AZtec version 3.0. https://nano.oxinst.com/products/aztec/.

Biofilms formed on the carbon steel coupons were observed under the field emission scanning electron microscope (FESEM) (Fig. 7). The micrograph of the coupon exposed to abiotic conditions revealed the presence of a thin layer of corrosion products derived from corrosion reactions promoted by components in the test solution (Fig. 7a). Micrographs of the three bacterial isolates showed their ability to form biofilms over the metal surface (Fig. 7b-d). Apart from bacteria cells, deposits and corrosion products were also observed on the metal surface. Thicker layers of corrosion products and cells were seen in biofilms of *S. chilikensis* and *E. roggenkampii.* The cross-sectional profile also showed differences in the distribution of layers and the elemental maps of deposits and corrosion products over coupons exposed to biotic conditions (Fig. 8). Samples exposed to *S. chilikensis* and *E. roggenkampii* showed thicker layers of corrosion products. The major elements detected in coupons exposed to the three isolates were oxygen and iron. Carbon was also abundant in all samples; however, since the mounting resin was carbon-based, it was not possible to discriminate carbon from biological material. Apart from these three elements, phosphorous was one of the major elements on coupons exposed to *E. roggenkampii*, and sulphur one of the major elements on coupons exposed to *S. chilikensis*.

## Discussion

Several seal rings from an in-service offshore oil production facility failed during hydrostatic testing due to corrosion. This testing is a common practice to assess piping systems integrity before service, during service, or for qualification to increase the operating pressure in in-service pipelines^45^. This investigation comprised the characterisation of the microbial community present in corroded seal rings and the recovery of cultivable microorganisms for MIC laboratory investigation. Previous studies have described the risk of MIC associated with poor hydrotesting practices^46–48^. This is because the water used for the test is taken from natural systems (sea, rivers, lakes), which have a high concentration and diversity of microorganisms that can contaminate the facility if the water is not effectively treated^2,47^. The primary issue with hydrotesting is that the water used is typically left stagnant in the system for extended periods. Whilst the test lasts approximately 10 h, the water used may remain in place for months, in some cases years^49,50^, promoting the development of biofilms under the stagnant conditions.

The role that bacteria played on the corrosion failure of the seal rings (root cause analysis) was not the focus of this research. This investigation aimed to characterise and isolate microorganisms from corrosion products and study their potential to instigate MIC of carbon steel. Results from this investigation indicate that the biocide treatment applied to seawater used to flood the pipe was not effective at controlling all viable microorganisms. DNA analysis of corrosion products revealed that the microbial community on the different sampled corroded seal rings was similar and dominated by marine microorganisms. Several of these microorganisms have previously been associated with hydrocarbon degradation^51–53^, which may explain their thriving in the pipeline during the stagnant period. Microorganisms related to thermophilic oil environments were not detected. This suggests that MIC was mainly triggered during seawater flooding and subsequent preservation, not during normal operation conditions. The dominance of *Pseudomonas* genus in different corroded seal rings supports the assumption that microorganisms participated in the corrosion phenomenon. This genus has been found ubiquitously in diverse environments, and its ability to survive with basic minimal nutrient requirements and to tolerate harsh conditions have allowed it to persist in urban and natural settings^54^. Moreover, several *Pseudomonas* species are naturally resistant to a variety of antimicrobial substances^55–57^, which help explain its dominance in the corroded seal rings despite the biocide treatment applied to the seawater. Although DNA-based next-generation sequencing (NGS) analysis cannot discriminate between live and dead bacteria, the recovery and cultivation of several bacterial species from corrosion products prove that corrosion products hosted living microorganisms.

In this investigation, nine bacterial species were isolated from the failed seal rings and identified with the 16S rRNA gene sequence. Microorganisms can be classified in different microbial groups, including sulphide producing prokaryotes, acid-producing bacteria, iron-oxidising bacteria, iron-reducing bacteria, nitrate reducing bacteria. Some of the isolates can be classified in two or more of these groups, which show the versatility and complexity of the microbial population living in the corroded steel. Corrosion of carbon steel by isolated bacteria was also evaluated in this study. Results demonstrated that all bacterial isolates were able to catalyse corrosion reactions under anaerobic conditions. Surface and corrosion analyses revealed that microorganisms increased general corrosion rates and pitting rates of carbon steel. Different isolates triggered different corrosion rates and patterns of attack (Figs. 3, 4). In the short term corrosion experiments, coupons exposed to *E. roggenkampii* CCC-SPP14 exhibited the highest corrosion rate while coupons exposed to *P. aeruginosa* CCC-IOB1 showed the highest pitting rate.

Three isolates (*P. balearica*, *E. roggenkampii*, and *S. chilikensis*) associated with different microbial groups (iron-oxidising bacteria, acid-producing bacteria and iron-reducing bacteria, respectively) were further studied under semi-batch conditions for longer exposure time. The behaviour of *P. balearica* CCC-IOB3 isolate was different for short term and long term experiments. Carbon steel exposed to this isolate in serum bottles under batch conditions (short term) experienced corrosion inhibition; however, the isolate induced corrosion of carbon steel in the longer-term reactor study where semi-batch conditions allowed the replenishment of test solution. These results provided evidence that corrosion inhibition by bacteria is not necessarily a characteristic of specific microorganisms but instead, it is likely the result of experimental conditions. Contradictory corrosive behaviour of bacteria has been previously reported. Miller et al.^58^ showed that depending on biofilm distribution, *Shewanella oneidensis* MR-1 could inhibit or enhance corrosion of steel. When steel was completely covered by the biofilm, the bacteria inhibited corrosion, likely due to O_2_ scavenging, whereas when steel was partially covered, corrosion was accelerated in the uncovered area. MIC inhibition has also been indicated for some species of the *Pseudomonas* genus^59,60^. Studies suggest that biopassivation of the steel is influenced by the production of extracellular polymeric substance (EPS) and biofilm structure that prevent corrosive species in the bulk solution from reaching the metal surface. Nonetheless, the formation of a uniform biofilm layer does not always prevent corrosion. It has also been demonstrated that biofilms can promote corrosion by the creation of differential concentration cells, alteration of anion ratios, generation of corrosive substances, and inactivation of corrosion inhibitors^61^.

### Proposed MIC mechanisms triggered by selected isolates

Microbial corrosion has predominantly been associated with localised corrosion; however, most often, MIC results in a combination of general and pitting corrosion. General corrosion is the uniform oxidation of the metal across its surface resulting in mass loss from the metal, whereas, pitting corrosion is localised, and although it may result in relatively minor mass loss, it typically results in wall penetration and loss of containment thus significantly reducing the service life of industry assets^62^. Differences in the deterioration pattern and corrosion measurements suggest variations in the corrosion mechanisms promoted by each isolate. *E. roggenkampii* stimulated significant general corrosion while *S. chilikensis* resulted in the highest localised corrosion and the most severe pitting attack.

*Shewanella* species are well known for their metabolic versatility to utilise a variety of electron acceptors, which include nitrate, nitrite, thiosulphate, elemental sulphur, iron (III), Manganese (III), fumarate, among others^58,63–65^. Additionally, *Shewanella* can use the metal as electron donor by secreting electron shuttles such as riboflavins, or by producing conductive filaments (nanowires)^66^. The broad metabolic capabilities of this genus suggest that these microorganisms can induce or accelerate corrosion through different mechanisms including EET-MIC (extracellular electron transfer MIC) and CMIC (chemical MIC)^62^. The expression of various metabolic pathways depends on the nutrients availability and environmental conditions. For example, in the present investigation, the test solution only contained thiosulphate and nitrate as electron acceptors which limited the metabolism of *S. chilikensis* CCC-APB5 to the anaerobic respiration of these molecules. However, after initiation of the corrosion process on the metal surface, the iron oxides layers produced could have also been used by the isolate as electron acceptors during the degradation of organic compounds. Likewise, the isolate could have induced corrosion by using the carbon steel as electron donor for the reduction of the thiosulphate, nitrate, and iron oxides. The genome sequence of *S. chilikensis* (GenBank accession number CP045857) confirmed that the isolate has the genetic machinery to conduct all these activities. A dual system for nitrate reduction (NAP, NAR), a system for metal reduction (MTR), genes involved in thiosulphate reduction (*phsA*, *glpE*), genes for riboflavin biosynthesis (*ribF*), and genes related to nanowires (prepilin peptidases and biogenesis proteins – Msh complex) were detected in its genome. *S. chilikensis* was the only isolate that produced sulphide and blackening of the test solution during the experimental period. The use of thiosulphate for anaerobic respiration was confirmed with the X-ray maps of the corrosion products that revealed the presence of iron and sulphur elements along the cross-sectional profile. During thiosulphate reduction, hydrogen sulphide is generated and in contact with ferrous iron precipitates as iron sulphide. Apart from sulphur related metabolism, *S. chilikensis* also expressed nitrate metabolism, which was determined by the presence of nitrite in the test solution after microbial growth and its absence in the control test. Hence, *S. chilikensis* could have contributed to MIC via three mechanisms, the dissolution of protective iron oxides, the production of corrosive metabolites (nitrite and H_2_S), and the uptake of electrons from the metal surface. It is important to highlight that *Shewanella* spp. are usually classified as iron-reducing bacteria (IRB); however, in this study, *S. chilikensis* reduced thiosulphate and nitrate, which again, demonstrates the versatility of these microbes.

*E. roggenkampii* CCC-SPP14 isolate belongs to the *Enterobactereacea* family, which is mostly comprised of acid-producing and nitrate reducing bacteria^67^. Some species of the *Enterobacter* genus have also been classified as iron-oxidising bacteria with the ability to cause MIC of carbon steel^68^. The capability of *E. roggenkampii* isolate to perform carbohydrate fermentation, iron oxidation and nitrate reduction was also confirmed through the genome sequencing (GenBank accession number JAACJF000000000). The pH decrease observed in the test solution suggested that *E. roggenkampii* was using the fermentation pathways and producing acids during its metabolic activities. The highest general corrosion observed in the coupons exposed to this isolate support the acid corrosion mechanism^46,69^. Although more than 90% of MIC occurs as pitting corrosion, it has been reported that the stimulation of acid corrosion by fermenting microorganisms promotes general corrosion^46^. Similar to the *S. chilikensis* experiment, nitrite was also detected in the test solution after microbial growth, indicating that the species reduced nitrate. Accumulation of nitrites could have induced the creation of the pits observed on the metal surface^70^. Based on the above, *E. roggenkampii* was likely to contribute to MIC via production of corrosive metabolites such as acidic species and nitrites.

The *Pseudomonas* genus has been reported several times to be involved in corrosion processes^38,71–74^. Species that belong to this genus are pioneer colonisers in biofilm formation^60^. It has been reported that the metal cation binding by EPS increases the ionisation of metals, leading to the accumulation of metal ions that change the electrochemical properties of the metal surface and promote corrosion^75^. Moreover, *Pseudomonas* species produce electron mediators such as pyocyanin and phenazine‑1‑carboxamide (PCM) that facilitate electron transfer between cells and metals, and accelerate corrosion rates by EET-MIC mechanism^76,77^. Most of the previous investigations of the corrosion abilities by *Pseudomonas* species have been carried out simulating aerobic environments^38,71,73,74^. Therefore, the primary corrosion mechanism described in the literature for *Pseudomonas* spp. is the formation of differential aeration cells. Some studies have also reported corrosion under anaerobic conditions and the described mechanism is iron oxidation coupled to nitrate reduction^72,78^. *P. balearica* CCC-IOB3 was isolated in IOB medium and precipitation of iron as a result of iron oxidation was observed in colonies grown on solid medium, which only contained nitrate as an electron acceptor. The presence of genes involved in iron metabolism and nitrate reduction was confirmed through genome sequencing (GenBank accession number CP045858)^79^. Different to the IOB medium, the test solution used for the corrosion evaluation had different nutrients (acetate, glucose, casamino acids), which the isolate could have used as electron donors for a heterotrophic nitrate reduction. Nonetheless, the metal surface (Fe^0^) or ferrous iron (Fe^2+^) released from the corrosion process could have also been used by this isolate as electron donors for nitrate reduction. High precipitation of iron oxides over the surface of the coupon was not observed in the FESEM analysis, suggesting that iron was not the main electron donor in the metabolism. Hence, the most likely MIC mechanism that promoted pitting of carbon steel in the presence of this isolate was the production of nitrite via nitrate reduction. Nevertheless, EET-MIC and iron oxidation cannot be disregarded entirely in this study.

The production of nitrite during anaerobic respiration was shared by the three isolates as a possible corrosion mechanism. Although nitrite is known as a corrosion inhibitor, it can also induce pitting corrosion when it is not available in a sufficient concentration that can fully passivate the steel^80^. It has also been reported that nitrite increases the ionic conductivity of the bulk fluid, which accelerates the corrosion rate^70^. Accumulation of nitrite is the most expected corrosion mechanism of nitrate reducing bacteria; however, other mechanisms induced by this microbial group have been proposed including the formation of concentration cells, the consumption of the cathodic H_2_ (cathodic depolarisation), and the direct microbial metabolism of Fe(0)^62^. Confirmation of all possible corrosion mechanisms triggered by the bacteria isolated in this study requires further investigation, including their behaviour under different environmental conditions and their effect on other steels.

## Conclusion

An investigation of corroded seal rings showed that the rings were inhabited by a microbial community dominated by members of the following genera *Pseudomonas*, *Martelella, Marinomonas, Shewanella, Alcanivorax,* and *Halomonas*, which have been associated with hydrocarbon degradation*.* In all sampled seal rings, *Pseudomonas* was the most abundant genus, which supported the hypothesis that microorganisms participated in the corrosion of the seal rings. Additionally, nine different microbial species were isolated from corrosion products and their ability to induce corrosion of carbon steel demonstrated in the laboratory. Differences in the surface deterioration patterns among isolates indicated variations in the corrosion reactions and mechanisms promoted by each isolate. Three isolates were further investigated to understand their effects on corrosion. Differences in the corrosion rates were observed among the isolates; *S. chilikensis* triggered the most severe pitting on carbon steel, followed by *E. roggenkampii*, and *P. balearica*. Corrosion mechanisms of the selected microorganisms were suggested based on their metabolic capabilities, which included the production of corrosive metabolites: organic acids, nitrites and H_2_S.

## Methods

### Sample collection and microbiological characterisation

#### Sampling

Samples were collected from corroded seal rings (made of 17–4 PH martensitic precipitation-hardening stainless steel) that failed during hydrostatic testing in the piping system of a FPSO facility located on the Australian North West Shelf. Corrosion products had a black/dark brown colour, containing both thick and thin solids. Corrosion products and sessile microorganisms attached to the deposits from three different corroded seal rings were scrapped and collected in sterile containers. Samples were transported at 4 °C to a research facility (2 days after collection) for microbiological analysis. Upon arrival, samples for microbial growth were processed immediately, and samples for DNA sequencing were stored at − 20 °C until extractions were conducted (maximum 2 weeks after collection).

#### Microbial composition analysis by 16S rRNA sequencing of DNA extracted from corrosion products of failed seal rings

The composition of the microbial communities on the failed seal rings was determined using Illumina NGS of the 16S rRNA gene hypervariable region V3–V4. For this, DNA was extracted from corrosion products using the DNeasy PowerSoil Kit (Qiagen), following the manufacturer’s instructions. DNA concentration was quantified fluorometrically with the Qubit dsDNA HS Assay kit (Life Technologies), and PCR reaction was carried out using the prokaryotic universal primers 341F (5′ CCTAYGGGRBGCASCAG 3′) and 806R (5′ GGACTACNNGGGTATCTAAT 3′)^81^, which have been previously applied for identification of microbial communities in corrosion products and oil production facilities^82,83^. Library preparation and sequencing were conducted by the Australian Genome Research Facility (AGRF), as described elsewhere^83^. Downstream analysis of the sequences was completed with QIIME2 version 2019.4^84^. The q2-dada2 plugin^85^ was applied for denoising and q2-feature-table plugin for the removal of singletons and low-frequency sequences (< 10 reads). Subsequently, taxonomy was assigned to each amplicon sequence variant (ASV) using the q2‐feature‐classifier plugin^86^ and q2-classify-consensus-blast plugin^87^. SILVA database version 132^88^ was used for the taxonomic classification. Microbial composition was visualised in bar charts created with the ggplot2 package version 3.1.0^89^ employing R version 3.4.3^90^. Only taxonomic orders with relative abundances equal or greater to 1% in at least one sample were included in the graph.

### Bacterial isolation, identification and phylogenetic analysis

#### Enrichment and isolation

Corrosion products were inoculated into several culture media to maximise the recovery of cultivable populations. Culture media targeted the growth of SPP, APB and IOB. The culture media for the recovery of IOB (iron oxidising bacteria media) and APB (phenol red dextrose broth) were prepared according to the guidelines and composition described in the standard test method NACE TM0194^91^. Composition of the culture medium used for the recovery of SPP is described elsewhere^92^. All culture media were prepared anaerobically and dispensed in Hungate tubes (15 mL capacity). In an anaerobic chamber filled with N_2_–CO_2_–H_2_ (75:10:5) gas mixture, 1 g of each sample was inoculated in the Hungate tubes containing 10 mL of growth medium. Tubes were incubated in dark for 28 days at 40 °C without shaking. After the incubation period, cultures that showed positive growth were subcultured onto solid media using the anaerobic chamber. Solid medium was prepared by adding 15 g L^−1^ of agar–agar (Sigma-Aldrich) to the same composition used to make the broths. Plate cultivation resulted in the growth of colonies with different morphologies; only one colony representative of each morphology type was selected for purification and further study. Purification was achieved using the streak plate method. All inoculated plates were incubated under anaerobic conditions by placing them in anaerobic jars with AnaeroGen sachets (Oxoid, Thermo Fisher Scientific). Single colonies were transferred to liquid medium, and microscopic observation in a phase contrast microscope (Nikon Eclipse Ci-L) was used to confirm consistent cell morphology of isolates.

#### 16S rRNA gene sequencing and identification of closest related species

Molecular identification of isolates was based on 16S rRNA gene sequencing. A 5 mL aliquot of a pure culture in an exponential growth phase was centrifuged at 15,000×*g* for 5 min to harvest cells from each isolate. Genomic DNA was extracted with DNeasy PowerSoil Kit (Qiagen), as described by the manufacturer. For PCR amplification, the universal bacterial primers 27F (5′ AGAGTTTGGATCCTGGCTCAG 3′)^93^ and 1492R (5′ CGGTTACCTTGTTACGACTT 3′)^94^ were employed. The reaction mixture was prepared according to the BIOTAQ DNA polymerase protocol (Bioline), with a final volume of 25 μL per reaction. Amplification was conducted in a T100 Thermal Cycler (Biorad) by the following steps: initial denaturation at 95 °C for 5 min, 35 amplification cycles of denaturation at 95 °C for 30 s, annealing at 55 °C for 30 s, and extension at 72 °C for 60 s, followed by a final extension at 72 °C for 10 min. PCR products were purified with the Wizard SV Gel and PCR Clean-Up System (Promega), and Sanger sequencing was conducted by AGRF. Sequences of 16S rRNA genes were compared with sequences stored in the NCBI using the standard nucleotide basic local alignment search tool (BLASTn)^95^.

#### Phylogenetic analysis

Phylogenetic and similarity analyses were conducted using the Molecular Evolutionary Genetics Analysis version 10 (MEGAX)^96^. ClustalW function was used for alignment of the 16S rRNA gene sequences found in this study and other organisms related retrieved from the NCBI. Alignment gaps were treated as missing data. The phylogenetic tree was constructed by the Neighbour-Joining method^97^, and 1,000 bootstrap replications were carried out to validate internal branches^98^. The paired similarity and pairwise distance were calculated using the transversion/transition weighting (*R* = *s*/V) and the Kimura-2-parameter model^99^.

### Corrosion experiments

#### Screening corrosion test of isolates

To evaluate their corrosive potential, all isolates were grown separately under anaerobic conditions using serum bottles (100 mL capacity) containing a metal sample. The metal used in this study was commercial 1,030 carbon steel, which is commonly used in the oil and gas industry. This material had the following elemental composition (weight %): C (0.43–0.5), Mn (0.6–0.9), Si (0.15–0.35), S (0.01–0.35), P (0–0.035), Cr (0–0.40), and Fe (balance). Square coupons (1 × 1 × 0.3 cm) with an exposed area of 1 cm^2^ were prepared for the analysis. First, coupons were electro-coated with a protective epoxy (Powercron 6000CX, PPG Industrial coatings). Then, one side of the coupon was wet ground (deionised water) using silicon carbide papers of 80, 120, 320, and 600 grit, consecutively. The polished specimens were washed with ethanol, dried with nitrogen gas and stored in a desiccator until the test. Before the test, coupons were sterilised with ultraviolet (UV) radiation for 15 min each side before immersion in the test solution. One coupon was immersed in each serum bottle and placed in the bottom at a horizontal position. Serum bottles were filled with 90 mL of test solution that had been prepared anaerobically by sparging a gas mixture of N_2_–CO_2_ (80:20). Supplemented artificial seawater was used as test solution, which had the following composition: 35 g L^−1^ of sea salts (Millipore), 20 mM of Na-lactate, 20 mM Na-acetate, 20 mM Na-formate, 10 mM glucose, 1.3 g L^−1^ bacto casamino acids (BD), 20 mM NH_4_NO_3_, 10 mM Na-thiosulphate, 1 mM iron (III) citrate, 1 mM iron (II) sulphate heptahydrate, and 1 mM Na-sulphide nonahydrate. The pH of the test solution was adjusted to 7.3 with sodium bicarbonate. Serum bottles were inoculated with 1 mL at an appropriate dilution of overnight cultures of the isolated bacteria to obtain a final concentration of 1 × 10^6^ cells mL^−1^ in each bottle. All isolates were evaluated independently and in triplicate by inoculation of the same culture in three serum bottles. Cultures were incubated for 7 days at 40 °C and shaking of 100 rpm. At the completion of the immersion period, coupons were removed and the surface examined for corrosion using light optical microscopy. Coupons in three serum bottles without the addition of bacteria were exposed to the same conditions to discriminate abiotic corrosion.

#### Additional corrosion experiments for selected isolates

Three isolates were selected and studied for a longer period of time (21 days) using glass reactors (150 mL capacity) under anaerobic conditions. Carbon steel round coupons with same elemental composition described earlier, and an exposed area of 1.27 cm^2^ were used for this experiment. A total of four metal coupons were immersed in each reactor. These metal coupons were prepared as described in “Screening corrosion test of isolates” section. Supplemented artificial seawater was used as test solution, which had the following composition: 35 g L^−1^ of sea salts (Millipore), 20 mM of Na-lactate, 20 mM Na-acetate, 30 mM Na-formate, 10 mM glucose, 1.3 g L^−1^ mM bacto casamino acids (BD), 15 mM NH_4_NO_3_, 8 mM Na-thiosulphate, 0.007 mM iron (II) chloride tetrahydrate, 0.007 mM manganese (II) chloride tetrahydrate, and 59 mM Na-bicarbonate. The pH of the test solution was 7.3 ± 0.2. Anaerobic conditions were maintained by continuous sparging of the test solution with a filter sterilised gas mixture of N_2_–CO_2_ (80:20) at a flow rate of 20 mL min^−1^. Each reactor was inoculated with one of the selected isolates to a final concentration of 1 × 10^7^ cells mL^−1^. The temperature was controlled at 40 °C ± 1 °C with an IKA RCT digital hotplate and a thermocouple. To maintain active growth of isolates during coupons exposure, the test reactors were operated under semi-batch conditions, by replenishing 70% of the test solution with fresh test solution every 3 days. After 21 days, the presence of sulphide and nitrites were determined using the sulphide methylene blue method 8,131 (Hach) and nitrate/nitrite semi-quantitative test strips (Quantofix). Coupons were analysed as described in the following section. A sterile control without the addition of bacteria was set up under the same conditions to discriminate abiotic corrosion.

### Analytical methods

#### Corrosion measurements

Corrosion measurements were carried out to determine corrosion rates and pitting on the metal surface. For this, three coupons from each test were cleaned by immersion in Clarke’s solution and sonication for 30 s, followed by washing steps with deionised water and ethanol. Cleaning cycle was repeated until a constant weight was measured with an electronic balance. Corrosion rates were estimated from weight loss, as described in the ASTM G1 standard^100^. Pitting analysis was conducted with a 3D optical profilometer (Alicona InfiniteFocus G4) using the instrument’s software (IFM version 3.5). Pitting rate was calculated using the maximum pit depth found in each test, as described in the NACE SP-0775 standard practice^44^.

#### Microbial quantification

Direct cell counts of planktonic (bulk solution) bacteria were performed periodically using Neubauer counting chamber and a phase-contrast microscope (Nikon Eclipse Ci-L). After 21 days of exposure, the number of bacteria attached to the metals (sessile) was determined by the qPCR method. Bacteria were detached from the coupons by vortexing and sonication in 10 mL of sterile 1 × PBS (Sigma-Aldrich) as described elsewhere^101^. Detached cells were harvested by centrifugation at 15,000×*g* for 5 min. The pellet obtained after centrifugation was used for DNA extraction and subsequent qPCR analysis. Genomic DNA was extracted using DNeasy PowerBiofilm Kit (Qiagen), as described by the manufacturer. The number of total bacteria was estimated by quantifying the number of copies of the *rpoB* gene, which is a single-copy gene^102^. PCR reaction was conducted as described elsewhere^83,103^. All analyses were performed in triplicate.

#### Biofilm imaging and surface analysis

One coupon from each reactor was examined using FESEM to visualise the morphology of the biofilm. The analysis was executed using a Neon Dual-Beam field emission scanning electron microscope (Zeiss). For the analysis, biofilms were prepared as described elsewhere^104^.

Additionally, a cross-sectional analysis of the corrosion products and biofilms was performed using the FESEM coupled with energy-dispersive X-ray spectroscopy (EDS). Coupons for this analysis were dried under nitrogen flow for 5 days. Then, coupons were mounted in Epofix Resin under vacuum (0.1 bar), followed by dry-polishing to 1 µm diamond finish to reveal the cross-sectional profile^105^. Cross-sectioned samples were coated with a platinum layer (5 nm thick) and observed in the microscope for elemental composition analysis. AZtec version 3.0 (Oxford Instruments NanoAnalysis) was used for FESEM/EDS data analysis.

#### Statistical analysis

Corrosion rates and measurements were performed in triplicate, and results are presented as the mean ± standard deviation. Kruskal–Wallis analysis was implemented to test if there were statistically significant differences in the corrosion rates between biotic and abiotic tests. All statistical analyses were performed with PAST version 3.25^106^ software. The statistical significance for all analyses was accepted at *p* < 0.05 (significance level).

### Sequence data deposition

The nucleotide sequences of the NGS data reported in this study were deposited in the NCBI under the bioproject number PRJNA613253. The 16S rRNA gene sequences of the isolated bacteria were deposited in the GenBank with the accession numbers MT215169, MT215170, MT215171, MT215172, MT215173, MT215174, MT215175, MT215176, and MT215177.

### Role of the funding source

The authors declare that Woodside Energy Ltd. contributed financial resources to assist this work via a postgraduate scholarship. The study sponsor has reviewed and approved the submission of the manuscript for publication.

## Supplementary information

Supplementary file 1.

## References

[1] Toba K, Suzuki T, Kawano K, Sakai J (2011). Effect of relative humidity on ammonium chloride corrosion in refineries. Corrosion.

[2] Machuca, L. L. Microbiologically influenced corrosion: A review focused on hydrotest fluids in subsea pipelines. In *ACA Corrosion and Prevention 2014,* Paper No. 117, Australasian Corrosion Association (Darwin, Australia, 2014).

[3] Wu S (2018). Environmental influence on mesh corrosion in underground coal mines. Int. J. Min. Reclam. Env..

[4] Machuca LL, Bailey SI, Gubner R (2012). Systematic study of the corrosion properties of selected high-resistance alloys in natural seawater. Corros. Sci..

[5] Cui J, Wang D, Ma N (2017). A study of container ship structures' ultimate strength under corrosion effects. Ocean Eng..

[6] Sun H, Shi B, Yang F, Wang D (2017). Effects of sulfate on heavy metal release from iron corrosion scales in drinking water distribution system. Water Res..

[7] Zumelzu E, Cabezas C (1996). Observations on the influence of cleaners on material corrosion in the food industry. Mater. Charact..

[8] Fergus JW (2013). Corrosion in nuclear waste containers. ECS Tran..

[9] Tait WS, Kutz M (2018). Electrochemical corrosion basics. Handbook of Environmental Degradation of Materials.

[10] Koch G (2016). International Measures of Prevention, Application, and Economics of Corrosion Technologies Study.

[11] Wolodko, J. *et al.* Modeling of microbiologically influenced corrosion (MIC) in the oil and gas industry—past, present and future. In *Corrosion 2018, Conference & Expo, Paper No. 11398* (NACE International, Phoenix, Arizona, 2018).

[12] Beavers JA, Thompson NG, Cramer SD, Covino BS (2006). External corrosion of oil and natural gas pipelines. ASM Handbook Volume 13C: Corrosion: Environments and Industries.

[13] Beech IB, Sztyler M, Gaylarde CC, Smith WL, Sunner J, Liengen T, Féron D, Basséguy R, Beech IB (2014). Biofilms and biocorrosion. Understanding Biocorrosion.

[14] Beech IB, Sunner J (2004). Biocorrosion: Towards understanding interactions between biofilms and metals. Curr. Opin. Biotechnol..

[15] Videla HA, Herrera LK (2005). Microbiologically influenced corrosion looking to the future. Int. Microbiol..

[16] Machuca LL, Polomka A (2018). Microbiologically influenced corrosion in floating production systems. Microbiol. Aust..

[17] Wade, S. A ., Mart, P. L. & Trueman, A. R. Microbiologically influenced corrosion in maritime vessels. In *ACA Microbiologically Influenced Corrosion Symposium,* (Australasian Corrosion Association, Melbourne, Australia, 2011).

[18] Mahoney DG, Marsh, MacLennan (1997). Large Property Damage Losses in the Hydrocarbon-Chemical Industries: A Thirty-Year Review.

[19] Sooknah, R., Papavinasam, S. & Revie, R. W. Validation of a predictive model for microbiologically influenced corrosion. In *Corrosion 2008, Conference & Expo, Paper No. 08503,* (NACE International, New Orleans, Louisiana, 2008).

[20] Abdullah A, Yahaya N, Md Noor N, Mohd Rasol R (2014). Microbial corrosion of API 5L X-70 carbon steel by ATCC 7757 and consortium of sulfate-reducing bacteria. J. Chem..

[21] Jacobson G (2007). Corrosion at Prudhoe Bay—A lesson on the line. Mater. Perform..

[22] Skovhus TL, Lee JS, Little BJ, Enning D, Skovhus TS, Lee JS (2017). Predominant MIC mechanisms in the oil and gas industry. MIC in the Upstream Oil and Gas Industry.

[23] Javed MA, Stoddart PR, Wade SA (2015). Corrosion of carbon steel by sulphate reducing bacteria: Initial attachment and the role of ferrous ions. Corros. Sci..

[24] Jia R, Yang D, Xu D, Gu T (2018). Carbon steel biocorrosion at 80 °C by a thermophilic sulfate reducing archaeon biofilm provides evidence for its utilisation of elemental iron as electron donor through extracellular electron transfer. Corros. Sci..

[25] Machuca LL, Lepkova K, Petroski A (2017). Corrosion of carbon steel in the presence of oilfield deposit and thiosulphate-reducing bacteria in CO_2_ environment. Corros. Sci..

[26] Gu T (2014). Theoretical modeling of the possibility of acid producing bacteria causing fast pitting biocorrosion. J. Microb. Biochem. Technol..

[27] Liu H, Gu T, Asif M, Zhang G, Liu H (2017). The corrosion behavior and mechanism of carbon steel induced by extracellular polymeric substances of iron-oxidising bacteria. Corros. Sci..

[28] Herrera LK, Videla HA (2009). Role of iron-reducing bacteria in corrosion and protection of carbon steel. Int. Biodeter. Biodegr..

[29] Xu D, Li Y, Song F, Gu T (2013). Laboratory investigation of microbiologically influenced corrosion of C1018 carbon steel by nitrate reducing bacterium *Bacillus licheniformis*. Corros. Sci..

[30] Uchiyama T, Ito K, Mori K, Tsurumaru H, Harayama S (2010). Iron-corroding methanogen isolated from a crude-oil storage tank. Appl. Environ. Microbiol..

[31] Head IM, Enning D, Skovhus TS, Lee JS (2017). Microorganisms in the oil and gas Industry. Microbiologically Influenced Corrosion in the Upstream Oil and Gas Industry.

[32] Høiby N, Bjarnsholt T, Givskov M, Molin S, Ciofu O (2010). Antibiotic resistance of bacterial biofilms. Int. J. Antimicrob. Ag..

[33] Broussard, Z., Tidwell, T. J., Paula, R. D. & Keasler, V. Determining biocide selection and dosage recommendation via planktonic and sessile kill studies and subsequent biofilm regrowth: a case study. In *Corrosion 2017, Conference & Expo , Paper No. 9802* (NACE International, New Orleans, Louisiana, 2017).

[34] Emerentiana, S. *et al.* A case study for MIC evaluation and mitigation in two Argentinian oilfields. In *Corrosion 2017, Conference & Expo, Paper No. 9476* (NACE International, New Orleans, Louisiana, 2017).

[35] Duncan K (2009). Biocorrosive thermophilic microbial communities in Alaskan North Slope oil facilities. Environ. Sci. Technol..

[36] Roalkvam I, Drønen K, Enning D, Skovhus TS, Lee JS (2017). Two case studies of corrosion from an injection water pipeline in the north sea: corrosion control due to operation management and high corrosion potential due to nitrate mitigation. Microbiologically Influenced Corrosion in the Upstream Oil and Gas Industry.

[37] Hashemi SJ (2017). Bibliometric analysis of microbiologically influenced corrosion (MIC) of oil and gas engineering systems. Corrosion.

[38] Yuan SJ, Pehkonen SO (2007). Microbiologically influenced corrosion of 304 stainless steel by aerobic *Pseudomonas* NCIMB 2021 bacteria: AFM and XPS study. Colloids Surf. B.

[39] Scarascia G (2019). Effect of quorum sensing on the ability of *Desulfovibrio vulgaris* to form biofilms and to biocorrode carbon steel in saline conditions. Appl. Environ. Microbiol..

[40] Aruliah R, Ting Y-P (2014). Characterisation of corrosive bacterial consortia isolated from water in a cooling tower. ISRN Corros..

[41] Zhong C (2018). Pan-genome analyses of 24 *Shewanella* strains re-emphasise the diversification of their functions yet evolutionary dynamics of metal-reducing pathway. Biotechnol Biofuels.

[42] Subedi D, Vijay AK, Kohli GS, Rice SA, Willcox M (2018). Comparative genomics of clinical strains of *Pseudomonas aeruginosa* strains isolated from different geographic sites. Sci. Rep..

[43] Papavinasam, S. Chapter 11—monitoring—external corrosion. In *Corrosion Control in the Oil and Gas Industry* 715–750 (Gulf Professional Publishing, 2014).

[44] SP0775 *NACE Standard Practice, Preparation, Installation, Analysis, and Interpretation of Corrosion Coupons in Oilfield Operations* (NACE International, 2013).

[45] Prasad, R. Chemical treatment options for hydrotest water to control corrosion and bacterial growth. In* Corrosion 2003, Conference & Expo, Paper No. 03572* (NACE International, San Diego, 2003).

[46] Javaherdashti R (2003). Enhancing the effects of hydrotesting on microbiologically influenced corrosion. Mater. Perform..

[47] Darwin, A., Annadorai, K. & Heidersbach, K. Prevention of corrosion in carbon steel pipelines containing hydrotest water—An overview. In *Corrosion 2010, Conference & Expo, Paper No. 10401* (NACE International, San Antonio, Texas, 2010).

[48] Machuca LL, Enning D, Skovhus TS, Lee JS (2017). Microbiologically induced corrosion associated with the wet storage of subsea pipelines (wet parking). Microbiologically Influenced Corrosion in the Upstream Oil and Gas Industry.

[49] Borenstein S, Lindsay P (2002). MIC failure of 304L stainless steel piping left stagnant after hydrotesting. Mater. Perform..

[50] Zhao K, Gu T, Cruz I, Kopliku A (2013). Investigation of microbiologically influenced corrosion in pipeline hydrotesting using seawater. Mater. Perform..

[51] Gerdes B, Brinkmeyer R, Dieckmann G, Helmke E (2005). Influence of crude oil on changes of bacterial communities in Arctic sea-ice. FEMS Microbiol. Ecol..

[52] Mnif S, Chamkha M, Sayadi S (2009). Isolation and characterisation of *Halomonas* sp. strain C2SS100—A hydrocarbon-degrading bacterium under hypersaline conditions. J. Appl. Microbiol..

[53] Baltar F (2018). Specific effect of trace metals on marine heterotrophic microbial activity and diversity: Key role of iron and zinc and hydrocarbon-degrading bacteria. Front. Microbiol..

[54] Skariyachan S, Sridhar VS, Packirisamy S, Kumargowda ST, Challapilli SB (2018). Recent perspectives on the molecular basis of biofilm formation by *Pseudomonas aeruginosa* and approaches for treatment and biofilm dispersal. Folia Microbiol. (Praha).

[55] Moradali MF, Ghods S, Rehm BHA (2017). *Pseudomonas aeruginosa* lifestyle: A paradigm for adaptation, survival, and persistence. Front. Cell. Infect. Microbiol..

[56] Vikram A, Bomberger JM, Bibby KJ (2015). Efflux as a glutaraldehyde resistance mechanism in *Pseudomonas fluorescens* and *Pseudomonas aeruginosa* biofilms. Antimicrob. Agents Chemother..

[57] Carvalho-Assef APDA (2010). IMP-16 in *Pseudomonas putida* and *Pseudomonas stutzeri*: potential reservoirs of multidrug resistance. J. Med. Microbiol..

[58] Miller RB (2016). Use of an electrochemical split cell technique to evaluate the influence of *Shewanella oneidensis* activities on corrosion of carbon steel. PLoS ONE.

[59] Suma MS (2019). *Pseudomonas putida* RSS biopassivation of mild steel for long term corrosion inhibition. Int. Biodeter. Biodegr..

[60] San NO, Nazır H, Dönmez G (2014). Microbially influenced corrosion and inhibition of nickel–zinc and nickel–copper coatings by *Pseudomonas aeruginosa*. Corros. Sci..

[61] Alasvand Zarasvand K, Rai VR (2014). Microorganisms Induction and inhibition of corrosion in metals. Int. Biodeter. Biodegr..

[62] Miller RB, Lawson K, Sadek A, Monty CN, Senko JM (2018). Uniform and pitting corrosion of carbon steel by *Shewanella oneidensis* MR-1 under nitrate-reducing conditions. Appl. Environ. Microbiol..

[63] Philips J (2018). A novel *Shewanella* isolate enhances corrosion by using metallic iron as the electron donor with fumarate as the electron acceptor. Appl. Environ. Microbiol..

[64] Beliaev AS, Saffarini DA (1998). *Shewanella putrefaciens mtrB* encodes an outer membrane protein required for Fe(III) and Mn(IV) reduction. J. Bacteriol..

[65] Burns JL, DiChristina TJ (2009). Anaerobic respiration of elemental sulfur and thiosulfate by *Shewanella oneidensis* MR-1 requires *psrA*, a homolog of the *phsA* gene of *Salmonella enterica* serovar typhimurium LT2. Appl. Environ. Microbiol..

[66] Jin Y (2019). Sharing riboflavin as an electron shuttle enhances the corrosivity of a mixed consortium of *Shewanella oneidensis* and *Bacillus licheniformis* against 316L stainless steel. Electrochim. Acta..

[67] Machuca LL, Jeffrey R, Melchers RE (2016). Microorganisms associated with corrosion of structural steel in diverse atmospheres. Int. Biodeter. Biodegr..

[68] Ashassi-Sorkhabi H, Moradi-Haghighi M, Zarrini G, Javaherdashti R (2012). Corrosion behavior of carbon steel in the presence of two novel iron-oxidising bacteria isolated from sewage treatment plants. Biodegradation.

[69] Beech, I. *et al.* Simple methods for the investigation of the role of biofilms in corrosion. In *Biocorrosion network* (European Federation of Corrosion, 2000).

[70] Lee DY, Kim WC, Kim JG (2012). Effect of nitrite concentration on the corrosion behaviour of carbon steel pipelines in synthetic tap water. Corros. Sci..

[71] Abdolahi A, Hamzah E, Ibrahim Z, Hashim S (2014). Microbially influenced corrosion of steels by *Pseudomonas aeruginosa*. Corros. Rev..

[72] Jia R, Yang D, Xu J, Xu D, Gu T (2017). Microbiologically influenced corrosion of C1018 carbon steel by nitrate reducing *Pseudomonas aeruginosa* biofilm under organic carbon starvation. Corros. Sci..

[73] Zhou, E., Li, H., Xu, D. & Wang, J. Accelerated corrosion of 2304 duplex stainless steel by marine *Pseudomonas aeruginosa* biofilm. In *Corrosion 2017, Conference & Expo, Paper No. 9326* (NACE International, New Orleans, Louisiana, 2017).

[74] Xu D (2017). Accelerated corrosion of 2205 duplex stainless steel caused by marine aerobic *Pseudomonas aeruginosa* biofilm. Bioelectrochemistry.

[75] Beech I, Hanjagsit L, Kalaji M, Neal AL, Zinkevich V (1999). Chemical and structural characterisation of exopolymers produced by *Pseudomonas* sp. NCIMB 2021 in continuous culture. Microbiology.

[76] Huang Y (2018). Endogenous phenazine-1-carboxamide encoding gene *PhzH* regulated the extracellular electron transfer in biocorrosion of stainless steel by marine *Pseudomonas aeruginosa*. Electrochem. Commun..

[77] Huang L (2020). Pyocyanin-modifying genes *phzM* and *phzS* regulated the extracellular electron transfer in microbiologically-influenced corrosion of X80 carbon steel by *Pseudomonas aeruginosa*. Corros. Sci..

[78] Jia R, Yang D, Xu D, Gu T (2017). Anaerobic corrosion of 304 stainless steel caused by the *Pseudomonas aeruginosa* biofilm. Front. Microbiol..

[79] Salgar-Chaparro SJ, Castillo-Villamizar G, Poehlein A, Daniel R (2020). Machuca LL 2020 Complete genome sequence of Pseudomonas balearica strain EC28: An iron-oxidising bacterium isolated from corroded steel. Microbiol. Resour. Announc..

[80] Videla HA, Herrera LK (2009). Understanding microbial inhibition of corrosion. A comprehensive overview. Int. Biodeter. Biodegr..

[81] Yu Y, Lee C, Kim J, Hwang S (2005). Group-specific primer and probe sets to detect methanogenic communities using quantitative real-time polymerase chain reaction. Biotechnol. Bioeng..

[82] Salgar-Chaparro SJ, Machuca LL (2019). Complementary DNA/RNA-based profiling: Characterisation of corrosive microbial communities and their functional profiles in an oil production facility. Front. Microbiol..

[83] Salgar-Chaparro SJ, Lepkova K, Pojtanabuntoeng T, Darwin A, Machuca LL (2020). Nutrient level determines biofilm characteristics and the subsequent impact on microbial corrosion and biocide effectiveness. Appl. Environ. Microbiol..

[84] Bolyen, E. *et al.* QIIME 2: Reproducible, interactive, scalable, and extensible microbiome data science. *PeerJ PrePrints* (2018). 10.1038/s41587-019-0209-9 PMC7015180 31341288

[85] Benjamin JC (2016). DADA2: High-resolution sample inference from Illumina amplicon data. Nat. Methods.

[86] Nicholas AB (2018). Optimising taxonomic classification of marker-gene amplicon sequences with QIIME 2’s q2-feature-classifier plugin. Microbiome.

[87] Camacho C (2009). BLAST plus : architecture and applications. BMC Bioinform..

[88] Quast C (2012). The SILVA ribosomal RNA gene database project: Improved data processing and web-based tools. Nucleic Acids Res..

[89] Wickham H (2016). ggplot2: Elegant Graphics for Data Analysis.

[90] R Core Team, R: A language and environment for statistical computing in *R Foundation for Statistical Computing* (Vienna, Austria 2014).

[91] TM0194 NACE Standard, Field monitoring of bacterial growth in oil and gas systems, (*NACE International*, 2014).

[92] Suarez EM, Lepkova K, Kinsella B, Machuca LL (2019). Aggressive corrosion of steel by a thermophilic microbial consortium in the presence and absence of sand. Int. Biodeter. Biodegr..

[93] Lane DJ, Stackebrandt E, Goodfellow M (1991). 16S/23S sequencing. Nucleic Acid Techniques in Bacterial Systematics.

[94] Gillan DC, Speksnijder AGCL, Zwart G, De Ridder C (1998). Genetic diversity of the biofilm covering *Montacuta ferruginosa* (Mollusca, Bivalvia) as evaluated by denaturing gradient gel electrophoresis analysis and cloning of PCR-amplified gene fragments coding for 16S rRNA. Appl. Environ. Microbiol..

[95] Altschul SF, Gish W, Miller W, Myers EW, Lipman DJ (1990). Basic local alignment search tool. J. Mol. Biol..

[96] Kumar S, Stecher G, Li M, Knyaz C, Tamura K (2018). MEGA X: Molecular evolutionary genetics analysis across computing platforms. Mol. Biol. Evol..

[97] Saitou N, Nei M (1987). The neighbor-joining method: a new method for reconstructing phylogenetic trees. Mol. Biol. Evol..

[98] Felsenstein J (1985). Confidence limits on phylogenies: An approach using the bootstrap. Evolution.

[99] Kimura M (1980). A simple method for estimating evolutionary rates of base substitutions through comparative studies of nucleotide sequences. J. Mol. Evol..

[100] ASTM, G., Standard practice for preparing, cleaning, and evaluating corrosion test specimens, (*ASTM International*, 2017).

[101] Salgar-Chaparro, S. J., Machuca, L. L., Lepkova, K., Pojtanabuntoeng, T. & Darwin, A. Investigating the effect of temperature in the community structure of an oilfield microbial consortium, and its impact on corrosion of carbon steel. In *Corrosion 2019, Conference & Expo, Paper No. 13343* (NACE International, Nashville, Tennessee, 2019).

[102] Ogier J-C, Pagès S, Galan M, Barret M, Gaudriault S (2019). *rpoB,* A promising marker for analysing the diversity of bacterial communities by amplicon sequencing. BMC Microbiol..

[103] Salgar-Chaparro SJ, Lepkova K, Pojtanabuntoeng T, Darwin A, Machuca LL (2020). Microbiologically influenced corrosion as a function of environmental conditions: A laboratory study using oilfield multispecies biofilms. Corros. Sci..

[104] Machuca LL (2014). Filtration–UV irradiation as an option for mitigating the risk of microbiologically influenced corrosion of subsea construction alloys in seawater. Corros. Sci..

[105] Albahri M (2019). X-ray micro-computed tomography analysis of accumulated corrosion products in deep-water shipwrecks. Mater. Corros..

[106] Hammer Ø, Harper DAT, Ryan PD (2001). Past: Paleontological statistics software package for education and data analysis. Palaeontol. Electron..

