# Supplementary material for: Carbon steel corrosion by bacteria from failed seal rings at an offshore facility
Source: Sci Rep. 2020 Jul 23;10:12287. doi: 10.1038/s41598-020-69292-5 (PMC7378185; doi:10.1038/s41598-020-69292-5)
Supplement: Supplementary file 1 [file 41598_2020_69292_MOESM1_ESM.pdf]

Supplementary Material

Carbon Steel Corrosion by Bacteria from Failed Seal Rings at an Offshore Facility

Silvia J. Salgar-Chaparro[a], Adam Darwin[b], Anna H. Kaksonen[c], Laura L. Machuca[a*]

[a] Curtin Corrosion Centre, WA School of Mines: Minerals, Energy and Chemical Engineering, Curtin University, Kent Street, Bentley, WA 6102, Australia

[b] Woodside Energy Ltd., Perth, 6000 WA, Australia

[c] Commonwealth Scientific and Industrial Research Organization (CSIRO), Land and Water, 147 Underwood Avenue, Floreat WA 6014, Australia

*** Correspondence:**

Laura L. Machuca

l.machuca2@curtin.edu.au

Content

Table S1. Microbial community composition in failed seal rings based on partial 16S rRNA gene sequencing

Table S2. Morphological characterisation of bacteria isolated from corroded seal rings

Table S3. Weight loss of coupons exposed to abiotic and biotic conditions for 7 days.

Table S4. *p*-value calculated from the Kruskal-Wallis test applied to evaluate differences in the corrosion rates and pit depths among coupons exposed to the isolated bacteria and the control for 7 days.

Table S5. Classification of corrosion rates triggered by isolated bacteria

Table S6. Weight loss of coupons exposed to abiotic and biotic conditions for 21 days.

Table S7. *p*-value calculated from the Kruskal-Wallis test applied to evaluate differences in the corrosion rates and pit depths among coupons exposed to the isolated bacteria and the control for 21 days.

Fig S1. Images of the carbon steel coupons exposed for 7 days to the bacteria isolated from corroded seal rings.

Figure S2. Test solution pH (A) and planktonic cell numbers (B) during the long term study. Replenishment of media is indicated by the dashed line.

Table S1. Microbial community composition in failed seal rings based on partial 16S rRNA gene sequencing

Taxa	Relative abundance (%)		
	S1	S2	S3
Actinobacteria			
Micrococcus	0.01		
Bacteroidetes			
Marinifilum	0.01		
Cyanobacteria			
Synechococcus CC9902			0.02
Epsilonbacteraeota			
Arcobacter	2.22	0.19	0.09
Firmicutes			
Bacillus	0.21	0.27	1.82
Oceanobacillus		0.19	0.03
Sporosarcina			0.37
Unclassified *Carnobacteriaceae*			0.36
Vallitalea	0.01		
Proteobacteria			
Brevundimonas		0.15	
Martelella	0.20	5.66	0.05
Labrenzia	1.48	24.67	0.07
Bradyrhizobium		0.05	
Nautella	0.02		
Unclassified *Rhodobacteraceae*	0.01		
Tistrella	0.35		
Desulfofaba	0.02		
Halodesulfovibrio	0.05		
Desulfuromusa	0.01		
Geoalkalibacter	0.11	0.17	
Idiomarina	0.03		
Shewanella	10.91		
Achromobacter	0.06		
Ralstonia		0.08	
Enterobacter	3.39	0.47	
Escherichia-Shigella	0.57	2.67	0.08
Alcanivorax	6.46		
Chromohalobacter			0.73
Halomonas	1.91		
Salinicola	0.02		
Marinomonas	0.11	18.49	
Acinetobacter	0.01	0.16	0.11
Alkanindiges	0.04		
Psychrobacter			1.36
Pseudomonas	65.89	46.73	94.91
Vibrio	5.87		
Stenotrophomonas		0.05	

Table S2. Morphological characterisation of bacteria isolated from corroded seal rings

Colony ID	Colony Colour	Colony form	Colony elevation	Colony margin	Gram	Morphology
CCC-APB1	white	circular	raised	entire	negative	rod
CCC-APB3	pale yellow	circular	raised	entire	negative	rod
CCC-APB5	pale orange	circular	raised	entire	negative	rod
CCC-SPP14	white	circular	raised	entire	negative	rod
CCC-SPP15	black	punctiform	raised	entire	negative	rod
CCC-IOB1	brown	circular	raised	entire	negative	rod
CCC-IOB3	metallic brown	circular	flat	entire	negative	rod
CCC-IOB9	orange	punctiform	raised	entire	negative	rod
CCC-IOB10	dark orange	circular	raised	entire	negative	rod

Table S3. Weight loss of coupons exposed to abiotic and biotic conditions for 7 days.

	Weight loss (mg)		
**Test**	**Coupon 1**	**Coupon 2**	**Coupon 3**
Control	4.6	4.5	4.3
CCC-APB1	4.4	6.5	5.8
CCC-APB3	16.1	5.1	13.8
CCC-APB5	10.6	11.8	10.6
CCC-SPP14	27.4	44.1	20.1
CCC-SPP15	3.9	4.6	3.1
CCC-IOB1	14.1	22.1	17.2
CCC-IOB3	2.4	1.6	2.8
CCC-IOB9	9.7	5.7	11.8
CCC-IOB10	14.0	5.1	7.9

Table S4. *p*-value calculated from the Kruskal-Wallis test applied to evaluate differences in the corrosion rates and pit depths among coupons exposed to the isolated bacteria and the control for 7 days.

Test 1	Test 2	***p* value**	
		**Corrosion rate**	**Pit depth**
Control	CCC-APB1	0.275	**0.007**
Control	CCC-APB3	**0.049**	**0.007**
Control	CCC-APB5	**0.049**	**0.007**
Control	CCC-SPP14	**0.049**	**0.007**
Control	CCC-SPP15	0.275	**0.008**
Control	CCC-IOB1	**0.049**	**0.007**
Control	CCC-IOB3	**0.049**	0.212
Control	CCC-IOB9	**0.049**	**0.007**
Control	CCC-IOB10	**0.049**	**0.008**

$p > 0.05$ = not significant

Table S5. Classification of corrosion rates triggered by isolated bacteria

ID	Corrosion rate categorisation*	Maximum pitting rate Categorisation*
CCC-APB1	Moderate	High
CCC-APB3	Severe	Severe
CCC-APB5	Severe	High
CCC-SPP14	Severe	Severe
CCC-SPP15	Low	Severe
CCC-IOB1	Severe	Severe
CCC-IOB3	Low	Low
CCC-IOB9	Severe	Severe
CCC-IOB10	Severe	Severe

*Categorization corresponds to the corrosion and pitting rate calculated after subtracting the corrosion and pitting rate measured in the abiotic control. Corrosion rate categories (mm/y) Low (< 0.025), Moderate (0.025-0.12), High (0.13-0.25), Severe (> 0.25). Pitting rate categories (mm/y) Low (< 0.13), Moderate (0.13-0.20), High (0.21-0.38), and Severe (> 0.38).

Table S6. Weight loss of coupons exposed to abiotic and biotic conditions for 21 days.

	Weight loss (mg)		
**Test**	**Coupon 1**	**Coupon 2**	**Coupon 3**
Control	1.9	2.2	1.5
CCC-IOB3	7.1	6.2	4.8
CCC-SPP14	11.8	8.8	10.6
CCC-APB5	7.6	7.7	6.3

Table S7. *p*-value calculated from the Kruskal-Wallis test applied to evaluate differences in the corrosion rates and pit depths among coupons exposed to the isolated bacteria and the control for 21 days.

Test 1	Test 2	*p* value	
		**Corrosion rate**	**Pit depth**
Control	CCC-IOB3	**0.049**	**0.004**
Control	CCC-SPP14	**0.049**	**0.004**
Control	CCC-APB5	**0.049**	**0.004**

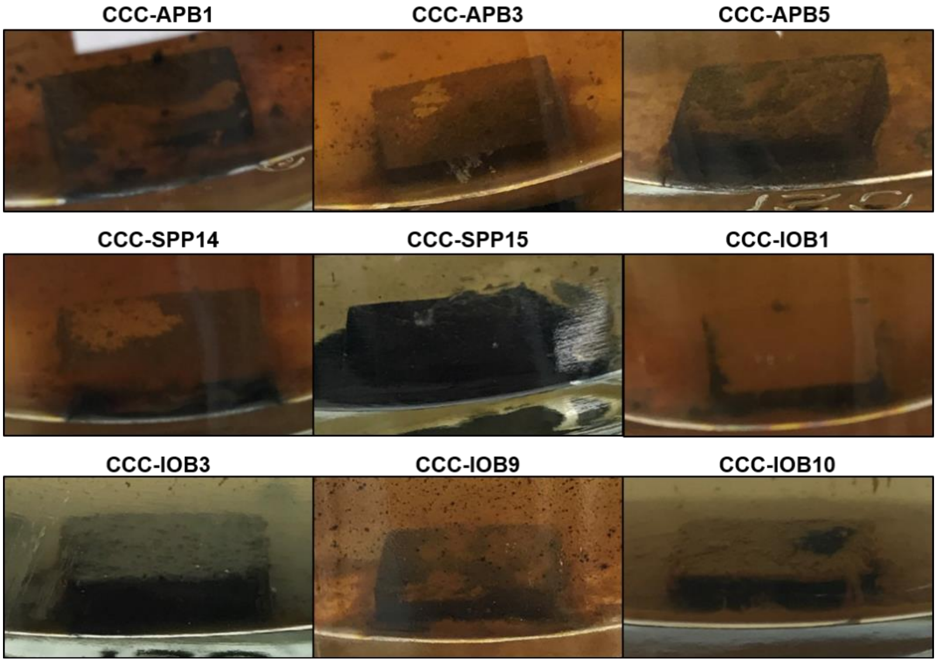

Figure S1. Images of the carbon steel coupons exposed for 7 days to the bacteria isolated from corroded seal rings.

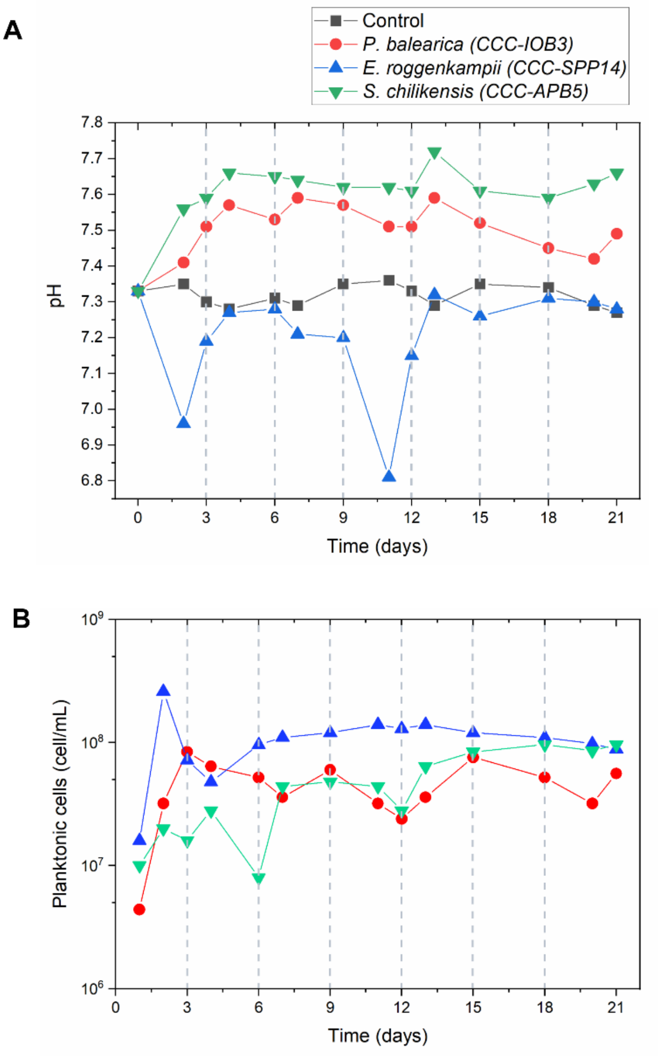

Figure S2. Test solution pH (A) and planktonic cell numbers (B) during the long term study. Replenishment of media is indicated by the dashed line. Figures generated using OriginPro version 2020 (OriginLab Corporation). https://www.originlab.com/
